# Medical Jurisprudence

**Published:** 1825-01-01

**Authors:** 


### MEDICAL JURISPRUDENCE.

*Medical Remuneration.** In the 15th Number of our last series (vol. iv. p. 545, *et seq.*) we took an opportunity of making some remarks on the degrading *mode* in which the general practitioner is remunerated for his professional services. We objected to the plan proposed by Mr. Alcock—that of charging for the *advice* and giving the *remedy* gratis, as being likely to occasion still more dissatisfation to the patient than the present mode, bad as this is. Mr. Greenhow is of the same opinion with ourselves, on this point. There is, unfortunately no plan which is free from objections. The most respectable plan would be to separate the dispensing of medicine from professional advice or chirurgical aid; but in any but large towns, we fear this is impracticable; and would, at all events, diminish the present revenue of the general practitioner (which is quite little enough) and throw the difference into the pockets of the chymists and druggists, who have already more than their share of pharmaceutical profits. The plan of prescribing without dispensing then must, we think, be abandoned for the present at least. The next most respectable plan (and we know that it is perfectly practicable) is to charge for attendance, and send in as little medicine as possible—and that at the same rate as the medicine procured at the chemists' shops. This measure, we have no doubt, might be effected by any individual, whether his brethren of the same town co-operated or not—for we have known it to be put in practice with perfect success. But unquestionably it would be far better for general practitioners to call meetings and bind themselves by solemn compact to pursue the plan unanimously, and then the public neither could nor would object. The example should be set in the metropolis and large towns. It is most degrading to send to patients, at the end of the year, a long bill, like that of a tailor or tallow-chandler. No item should ever be stated, except the two heads of medicine, and professional attendance. If, as might happen on some rare occasions, the patient demanded a sight of the separate debits, he should be shown the surgeon's ledger—but, we repeat it, no bill of parcels should ever be made out. This, we imagine, (and not without mature consideration) is the only amelioration of which the *mode* of remunerating the general practitioner is at present susceptible. But this *improvement* would be one of incalculable advantage to both patient and practitioner. Without altering the sum total of expence attending an illness, it would save the patient many a nauseous draught, and prevent many a *bitter* reflection on the rising pile of empty phials which so often forms an eye sore to the sick man in one corner of his room. It would shelter the practitioner from many illiberal

* Hints towards the adoption of an improved principle of remunerating the Surgeon-Apothecary. By T. M. Greenhow, Surgeon. 8vo. pp. 26.

suspicions respecting the *motive* of sending in so much medicine—and it would probably save him many unnecessary and fatiguing visits, when it was known that the visits would be charged to the patient's account. These are only a very few of the advantages attending the plan recommended. There are objections to the present *mode* of remuneration, which we touch upon with reluctance, and not without some degree of horror. We much fear that many *lives* annually fall a sacrifice to this degrading and mischievous system. Numerous facts have fallen within our own notice, which indeed, leave no doubt in our own minds on the subject. Many patients tamper with their complaints, or apply to the chymist, for fear of the apothecary's long bill and load of medicines, till the disease acquires a foundation that cannot afterwards be shaken by the most consummate skill. Many other patients deceive themselves and their medical attendants by throwing one half of the medicine into the fire; and thus plans of treatment are rendered abortive that were laid with judgment. And who will aver, that many stomachs are not rendered incapable of retaining and assimilating a *proper quantum* of medicinal agents, by the improper or unnecessary quantum of menstruum in which they are ordered to be swallowed? Every medical man, who has had the misfortune to be confined on the bed of sickness himself, will be able to answer this question. For our own parts, we are thoroughly convinced that many lives are daily lost in this way by the unhallowed mode of medical remuneration at present in use!

We object to Mr. Alcock's proposal that the general practitioner's fee, whatever it may be, should be paid at each visit. The fee must necessarily be so small (for we do not think it can average more than five shillings, and often less) that there would be something ludicrous as well as inconvenient in this procedure. The scheme proposed by the author before us is rather more feasible, but still, we think, objectionable—that of the practitioner receiving his fees whenever they amounted to a guinea. Even this plan would be too complicated for general practice. Mr. Greenhow's proposals, however, shall be given in his own words.

"1st. That the general practitioners in every town should hold meetings among themselves for the purpose of deciding on what they may consider an equitable scale of fees, both for their visits in town and in the country—the latter being regulated according to the distance. And that they should also form a table of prices for their medicines, on such terms as would no more than suffice to remunerate them for the expence of supplying them to their patients. But as it would be only fair that, in case of their being required to prepare prescriptions for patients, whom they might not themselves professionally visit, they should derive an adequate profit from the stock so employed, a second table of prices, regulated accordingly, would be required for such occasions.

"2d. That having formed resolutions agreeable to the preceding proposition, they should then call a general public meeting to ratify their proceedings, that the adoption of the improved system might become the joint act of the Public and of the Profession. A clear and candid statement of their views should be laid before that meeting, and it should be their endeavour to convince those present of the justice and reasonableness of the reform which it is intended to bring about.

The writer is sanguine enough to believe that a simultaneous movement amongst the Members of the Profession in every part of England, in the way above described, would not fail to be attended with all the success which the importance of the object deserves. He has only further to remark, that were the Members of the Profession in different towns to communicate freely with each other on the subject, it might lead to the very desirable result of establishing a scale of charges and of fees, uniform and well understood throughout the kingdom." 26.

With the exception already stated, we entirely agree with Mr. Greenhow—and we earnestly exhort the general practitioners of the metropolis to set about the work of reformation, by calling a general meeting—adopting such plans as their wisdom may suggest—and then making these plans and the reasons for their adoption extensively known throughout society at large through the medium of the press.

